# Exploring the Genetic Etiology of Trust in Adolescents: Combined Twin and DNA Analyses

**DOI:** 10.1017/thg.2016.84

**Published:** 2016-12

**Authors:** Robyn E. Wootton, Oliver S. P. Davis, Abigail L. Mottershaw, R. Adele H. Wang, Claire M. A. Haworth

**Affiliations:** 1 School of Experimental Psychology, University of Bristol, Bristol, UK; 2 MRC Integrative Epidemiology Unit, School of Social and Community Medicine, University of Bristol, Bristol, UK

**Keywords:** trust, twin design, heritability, DNA-based heritability

## Abstract

Behavioral traits generally show moderate to strong genetic influence, with heritability estimates of around 50%. Some recent research has suggested that trust may be an exception because it is more strongly influenced by social interactions. In a sample of over 7,000 adolescent twins from the United Kingdom’s Twins Early Development Study, we found broad sense heritability estimates of 57% for generalized trust and 51% for trust in friends. Genomic-relatedness-matrix restricted maximum likelihood (GREML) estimates in the same sample indicate that 21% of the narrow sense genetic variance can be explained by common single nucleotide polymorphisms for generalized trust and 43% for trust in friends. As expected, this implies a large amount of unexplained heritability, although power is low for estimating DNA-based heritability. The missing heritability may be accounted for by interactions between DNA and the social environment during development or via gene–environment correlations with rare variants. How these genes and environments correlate seem especially important for the development of trust.

Over a decade ago, the first law of behavioral genetics was asserted: ‘All human behavioural traits are heritable’ (Turkheimer, 2000, p. 160). This statement was based upon repeated findings that regardless of the behavior there was genetic influence to some degree. Recently, a large meta-analysis of twin studies showed average heritability of 49% across 17,804 traits, including measures of behavior, depression, anxiety, and personality (Polderman et al., 2015). Even ‘environmental’ influences, such as parenting, social support, and divorce, typically show genetic influence; average heritability of these measures was 27% in a meta-analysis (Kendler & Baker, 2007; McGue et al. 2014). However, recent research has suggested the trait of trust might be an exception to the finding of moderate heritability (Van Lange et al., 2014).

Definitions of trust vary across disciplines (McKnight & Chervany, 2001; Nannestad, 2008), with the broadest being *generalized trust*: the extent to which we trust people in general (Nannestad, 2008; Van Lange, 2015). Trust is an important component of social interactions on which our relationships are built and strengthened (McKnight et al., 1998; Van Lange, 2015). Trust is necessary to facilitate successful cooperation (Balliet & Van Lange, 2013; Jones & George, 1998; Nannestad, 2008) and strengthen social networks (Buskens, 2002; Nannestad, 2008). When trust is misplaced and broken, this damages relationships (Robinson, 1996). The benefit of cooperation to social evolution implies a genetic component is plausible (McNamara et al., 2009). Furthermore, understanding the genetic and environmental influences on trust could have implications for societal interventions. Increased social support and stronger social networks increase psychological well-being (Billings & Moos, 1981; Turner, 1981). With trust playing an important role in establishing and maintaining these relationships, understanding the etiology of trust might enable us to promote not just trust itself, but well-being more broadly, and to understand the underlying biological pathways.

## Genetic Influences on Trust

Previous genetically informative studies of trust have estimated it to be between 10% and 31% heritable (Cesarini et al., 2008; Hiraishi et al., 2008; Sturgis et al., 2010; Van Lange et al., 2014). The latest of these studies used an extended twin design to conclude that the genetic influence on trust in an adult sample was ‘virtually absent’ (Van Lange et al., 2014). Instead, the authors suggested that it was due to environmental factors, including cultural transmission. Cultural transmission is the passing of environments from parents to offspring: If people we trust cooperate, we learn to be trusting, and conversely, if people abuse our vulnerability, we learn to be cautious. Therefore, trust is more determined by social attributes than personal ones. Social explanations are rooted in attachment theory; our ability to trust is affected by early bonding experiences (Bowlby, 2005). With trust being governed by our relationships with other people and the social interactions we encounter, could it be predominantly socially influenced? Here, we estimate the importance of genetic and environmental influences on trust in an adolescent sample.

## The Current Study

Our first aim was to investigate the genetic influences on two different aspects of trust using a twin design. These two aspects were captured by different measures. The first captures generalized trust: our general beliefs about the nature of strangers. The second measures trust in friends and our already established attachments.

The second aim of this study was to calculate DNA-based heritability estimates of trust using genomic-relatedness-matrix restricted maximum likelihood (GREML) modeling. This method uses the twins’ genotyped common single nucleotide polymorphisms (SNPs) to account for variance in the phenotype of trust. Variance explained using this method can only be attributed to common genetic differences and their environmental correlates. Previous estimates of the related trait of subjective well-being estimate 4% of the variance can be explained by DNA-based heritability (Okbay et al., 2016). Consequently, it is hypothesized that there will be a modest heritable component to trust, partially explained by common genetic variants.

The third aim of this study was to identify specific SNPs associated with trust that partially explain the presence of DNA-based heritability. For this, we will use previously associated candidate genes. The SNP rs53576 found in the oxytocin receptor (OXTR) gene has previously been associated with trust (Nishina et al., 2015). SNPs associated with the phenotype agreeableness will also be investigated. Agreeableness and trust are phenotypically related traits because agreeableness is conceptualized as willingness to cooperate (Denissen & Penke, 2008) and trust is a component of the agreeableness dimension, as defined by McCrae and Costa (1997). It is hypothesized that these candidate SNPs will be significantly associated with the trait of trust and partially account for the DNA-based heritability.

## Methods

### Sample

The sample was taken from the Twins Early Development Study (TEDS), a cohort of British twins born between 1994 and 1996 in England and Wales. Data on trust were collected via postal questionnaire and online questionnaire in two studies when the twins were 16 years old; 3,864 families took part in the mailed assessments, which included the measure of generalized trust, and 6,248 families took part in the web assessments, which included the measure of trust in friends. The TEDS sample is representative of the overall UK population (Haworth et al., 2013). Families were excluded from analysis if they had not provided consent at first contact, did not meet medical exclusion criteria, had experienced perinatal complications, or were of unknown zygosity. Consequently, for assessing generalized trust, twin analyses were conducted on 7,352 twins, including 3,592 complete twin pairs (46% male and 54% female; 36% monozygotic twins (MZ) and 64% dizygotic twins (DZ)) with a mean age of 16.15 years. For assessing trust in friends, the sample consisted of 4,679 twins, including 2,069 complete twin pairs (41% male and 59% female; 39% MZ and 61% DZ) with a mean age of 16.47 years. Overall, the five twin categories (comprising sex and zygosity differences) were of the expected proportions (see Table S1).

Zygosity was determined using a questionnaire (Goldsmith, 1991) completed by parents at first contact and when the twins were 3 and 4 years of age. Genetic data found the questionnaire to be 95.7% accurate (Price et al., 2000). In cases where zygosity was uncertain, DNA markers were tested.

### Trust Measures

Two aspects of trust were measured: generalized trust and trust in friends. Generalized trust was assessed using the single item measure from the Gallup poll: ‘In general I think people can be trusted’. Responses were given on a dichotomous scale, either yes or no. This item is taken from the Gallup World Poll (2016) used to assess global well-being. Responses were collected via a booklet, completed individually by the twins. Trust in friends was assessed using the attachment subscale of the Inventory of Parent and Peer Attachment (IPPA; Armsden & Greenberg, 1987). This subscale consists of 10 items, assessed on a five-point Likert scale from *almost never or never true* to *almost always or always true*. Items included: ‘I trust my friends’ and ‘I can count on my friends when I need to get something off my chest’. We created the composite Trust in Friends scale by creating a cumulative score of the 10 items, requiring at least 50% of the items to be non-missing. This scale is reliable for use on adolescents, with Cronbach’s alpha = 0.92 in our sample. IPPA responses were collected as part of a battery of online measures.

### Genotyping

DNA samples were collected via buccal swabs. Genotyping of 3,665 unrelated individuals (only one per twin pair) was conducted using Affymetrix GeneChip 6.0 genotyping arrays as part of the Wellcome Trust Case Control Consortium 2 (http://www.wtccc.org.uk/ccc2/), resulting in around 700,000 high-quality genotyped SNPs. After quality control, 3,152 individuals left (46% male and 54% female). For details of genotyping and quality control, see Davis et al. (2014). Of these genotyped individuals, 1,656 had generalized trust scores, 1,115 had scores for trust in friends, and 560 had both.

## Statistical Analyses

### Twin Modeling

Trust variables were regressed against age and sex and all analyses were conducted using the OpenMx package (Boker et al., 2011) for R (R Core Team, 2014).

Twin analyses began by estimating correlations using a fully saturated model. Equating means and variances across twin pair and zygosity did not worsen fit for either measure of trust. The twin model assumes 100% genetic similarity for MZ twins and 50% for DZ twins. Consequently, differences in trust between MZ and DZ twin pairs can be used to decompose the variance into additive genetic, non-additive genetic, shared environmental, and non-shared environmental components, known as A, D, C, and E. In a simple twin design, it is not possible to model all four components at once as C and D are confounded. Therefore, we will use both ACE and ADE models.

A is the measurement of additive genetic variance. This is the extent to which genes contribute in a summative way to the trait of trust. C is shared environmental influence. Shared environmental effects serve to make the twins more similar to each other. E is non-shared environmental influences. These are anything that both twins experience differently and serve to make the twins less similar. The final component (which can only be estimated in place of C) is D, which represents non-additive genetic variance. These effects occur when the contributing genetic variants do not combine in an additive way to generate the variance seen in the trait. This could be for many reasons including dominant genes, recessive genes, or epistasis. The presence of D is indicated by a DZ correlation, which is less than half the MZ correlation.

### DNA-Based Heritability

GREML was conducted using the latest released version 1.25 of genome-wide complex trait analysis (GCTA; Yang et al., 2011). Analysis began with the construction of a genetic relationship matrix (GRM) containing the genetic relatedness for every pair of individuals. Relatedness was calculated using the SNP markers available from genotyping (direct genotyping only, imputed SNPs were not used; Speed et al., 2012). Anyone more genetically similar than fourth cousins (relatedness of > 0.195%) was removed from the analysis. Residual maximum likelihood modeling estimated genetic and residual components from the pairs’ genetic and trait similarity (Yang et al., 2011). Sex, age, and 10 principal components of population structure were covariates in all analyses.

### Replicating Previous SNP Associations

The SNP rs53576 found in the OXTR gene has previously been associated with trust (Nishina et al., 2015); however, this SNP was not available in the TEDS sample. Therefore, seven SNPs in LD with rs53576 (on chromosome 3) were used instead. A SNP located in the CD38 gene was also investigated as this is associated with oxytocin release (Jin et al., 2007). Eight SNPs previously associated with agreeableness were used (Kim et al., 2013; Terracciano et al., 2010). For genotype at each SNP, a linear regression was run against both phenotypes using the PLINK version 1.9 software (Purcell et al., 2007).

## Results

### Twin Modeling

The mean scores for generalized trust and trust in friends were 0.83 (*SD* = 0.37) and 32.27 (*SD* = 6.70), respectively, the first being a dichotomous score of either 0 or 1 and the second a cumulative score of 10 items, rated on a four-point scale, with a maximum total score of 40. The two measures were correlated (*r* = 0.25, *p* < .001). This relatively low correlation reflects that they are testing fundamentally different aspects of trust.

Generalized trust levels differed significantly by gender, with males (mean = 0.86, *SD* = 0.35) rating themselves as more trusting than females (mean = 0.81, *SD* = 0.39), *t*(7332.84) = 5.64, *p* < .001. Trust in friends also differed by gender, this time with females (mean = 32.86, *SD* = 6.91) rating themselves as more trusting than males (mean = 31.41, *SD* = 6.28), *t*(4316.58) = 7.43, *p* < .001. This suggests that males consider themselves slightly more willing to trust a stranger and females more able to confide in friends. However, the effect sizes for these sex differences were small. A regression procedure for age and sex corrected for mean effects before twin analysis (McGue & Bouchard, 1984).

Intraclass correlations are given in Table 1. Using OpenMx, we ran ACE and ADE models along with nested AE, CE, DE, and E models. Chi-squared tests and AIC estimates indicated that ADE was the best fitting model for both generalized trust and trust in friends (see Table 2). The standardized parameter estimates are given in Table 3. Two estimates of heritability can be given: The narrow sense estimate of heritability (only taking into account A) was 0.35 and 0.14 for generalized trust and trust in friends, respectively, and broad sense heritability estimates (taking into account A and D) were 0.57 and 0.51, respectively.

### DNA-Based Heritability

For generalized trust, the proportion of variance explained by common SNPs is 0.07 (*SE* = 0.17). This explains 20.88% of the narrow sense heritability twin estimate and 12.82% of the broad sense heritability twin estimate. However, this estimate does not differ significantly from 0 (*p* = .33). For trust in friends, the proportion of variance explained by common SNPs is 0.06 (*SE* = 0.24). These DNA-based heritability estimates account for 43.05% of the narrow sense twin heritability and 12.05% of the broad sense heritability. Again, this estimate is not significantly different from zero (*p* = .40); however, we note that the upperbound DNA-based heritability estimates for both generalized trust and trust in friends would explain a substantial proportion of the twin heritability. A larger sample size would provide more accurate estimates of the DNA-based heritability.

### Replicating Previous SNP Associations

None of the previously associated SNPs were significantly associated (at a lenient uncorrected *p* value of .05) in this sample for either generalized trust or trust in friends (see Table 4). For completeness, two genome-wide association studies (GWAS) were also conducted for generalized trust and trust in friends. For this analysis, the genome wide level of significance (*p* = 5 × 10^−8^) was used and no significant SNPs were identified as would be expected, given our sample size was underpowered for detecting small effects. Further information, Manhattan plots, and top SNPs are available in supplementary materials.

## Discussion

The findings from our twin analysis indicate a strong genetic influence on trust, with broad sense heritability estimates of 57% and 51% for generalized trust and trust in friends, respectively. This supports previous genetically informative investigations of trust, with estimates ranging from 10% to 31% (Cesarini et al., 2008; Hiraishi et al., 2008; Sturgis et al., 2010). For both measures, the best fitting model included a combination of additive and non-additive genetic effects. We also aimed to explain this variance using common genetic variants. Common genetic variants accounted for 21% and 43% of the modest additive genetic variance found for generalized trust and trust in friends, respectively. However, the large broad-sense heritability found in the twin study was largely unaccounted for by common genetic variation, as expected (13% and 12%, respectively, for generalized trust and trust in friends).

### Twin Results for Trust

Previous heritability estimates of trust range from virtually absent (Van Lange et al., 2014) to 31% (Hiraishi et al., 2008). This study found similar additive genetic estimates of 35% and 14%. Broad sense heritability estimates, accounting for non-additive genetic variance, were greater than all previous estimates at 57% and 51%. The source of this variability could be accounted for by measurement, sample or culture. First, measurement of trust varied between studies. All studies (except Cesarini et al., 2008) used questionnaires, but no two were the same. Hiraishi et al. (2008) used a general trust scale (Yamagishi, 1986) and the trust subscale items from the NEO Personality Inventory Revised (NEO-PI-R), with the questions in the latter case translated into Japanese. Sturgis et al. (2010) assessed political trust, and Van Lange et al. (2014) divided trust into trust-in-others and trust-in-self. Their measures assessed extreme attitudes, with items including ‘I dare to put my fate in the hands of most other people’. Asking for such a strong response from participants is potentially testing something different from our measure of generalized trust.

Second, sample sizes varied from the smallest, 146 pairs (Sturgis et al., 2010), to the largest, 530 pairs (Hiraishi et al., 2008). A larger sample gives the power to detect shared environmental influences (C) or non-additive genetic effects (D), and additive genetic variance (A). The present study, using over 2,000 pairs of twins, strongly indicated the presence of non-additive genetic effects with power unavailable in other samples.

Third, the age of the samples varied from the youngest in our sample (16 years of age) to the oldest, an average age of 45.3 years (Van Lange et al., 2014). It would be interesting to investigate the extent to which age accounts for the variability in estimates. Genetic and environmental influences do vary with age (Haworth & Davis, 2014); for example, heritability of intelligence and body mass index (BMI) increase (Haworth et al., 2008; Haworth et al., 2010) due to gene-environment correlations and our freedom to choose environments (Briley & Tucker-Drob, 2013). Future research following up heritability of trust with the same twin sample longitudinally could inform this further.

Finally, levels of trust vary with culture, as evidenced by the World Values Survey (http://www.worldvaluessurvey.org/wvs.jsp). When asking people from almost 100 countries around the world ‘Can most people be trusted?’, responses ranged from 60% affirming in China to 15% in Azerbaijan. Heritability of trust also varies with culture; estimates are reported to be absent for the Dutch sample (Van Lange et al., 2014), 10% in an American sample (Cesarini et al., 2008), 14–31% in an Australian sample (Sturgis et al., 2010), 20% in a Swedish sample (Cesarini et al., 2008), and finally 31% heritable in a Japanese sample (Hiraishi et al., 2008). These estimates are all lower than the broad sense heritability estimates of around 50% in our British adolescent sample. It is not clear whether these cross-country differences in heritability are due to the measures used in each study or to differences in the underlying influences on trust. Future studies should aim to use unified and comprehensive measures of trust.

### DNA-Based Heritability of Trust

Common genetic variants were found to account for only 12–13% of the broad sense heritability estimates from the twin study. One explanation for the discrepancy between the twin estimate of heritability and the DNA-based estimate of heritability is that the true genetic variation is the result of rare genetic variants that are not tagged. This would explain the gap between twin heritability and DNA-based heritability estimates. This is consistent with research suggesting that despite the high heritability estimates of subjective well-being, common variants have been found to explain only a very small amount, around 0.02–0.035% of the variance (Okbay et al., 2016). Subjective well-being is traditionally assessed using measures of life satisfaction and subjective happiness, which correlate on average 0.41 with the measures of trust in our sample. Meta-analyses of twin studies estimate the heritability of subjective well-being at 36% (Bartels, 2015) and DNA-based heritability estimates at only 4% (Okbay et al., 2016). Our results suggest that measures of trust may show a similar genetic architecture to measures of subjective well-being.

DNA-based heritability estimates are not immune to inflation from environments that are correlated with genetics (Davis et al., 2014). However, unlike twin-based heritability estimates, only environments that correlate with common SNP variants will bias the estimate, not all genetic variation. The same is true for cultural transmission. In a twin model, inability to estimate the effects of cultural transmission would bias the genetic component, causing it to appear larger than genetics would alone (Boomsma et al., 2002; Fulker, 1988). Van Lange et al. (2014) would have estimated 0.16 and 0.17 heritability for trust-in-others and trust-inself, respectively, if they had been unable to estimate cultural transmission. DNA-based heritability estimates will also be inflated by cultural transmission to the extent that the influence of the parental phenotype is correlated with the common SNPs inherited. Therefore, cultural transmission that correlates with rare variants and unmeasured genetic variation could be a second explanation for the missing heritability observed.

Beyond a simple biological pathway explanation (from genes, to brain, to behavior), gene-environment correlation is another potential pathway through which genes could associate with the trait of trust. Gene-environment correlation might be of particular importance for a trait as socially determined and complex as trust (Van Lange, 2015). However, a much greater sample size would first of all be needed to provide a more accurate estimate of DNA-based heritability.

### Replicating Previous SNP Associations

Attempts to replicate previous SNP associations were all unsuccessful. This could indicate that the previously associated SNPs were in fact false positives, common for candidate gene studies (Flint & Munafò, 2013). Our GWAS revealed no significant associations either, but this fits with the architecture of a complex trait. Trust is most likely polygenic, caused by many genes of small effects. Due to constraints on those who had provided both DNA samples and trust measures, a relatively small sample remained (*N* = 1,656 for generalized trust and 1,115 for trust in friends). For a complex trait, this is underpowered to detect SNPs of small effect. The sample size is also small with respect to DNA-based heritability analysis. Therefore, caution is advised in interpreting these results until further replication can be conducted in a larger sample.

### Limitations

As mentioned, the field of trust research would benefit greatly from unified measurement and definitions (Nannestad, 2008). For generalized trust, our single-item measure is not ideal. More items and response categories would increase reliability. Seeing as most people responded that they were trusting, a scale response would have given us more variation. However, both this measure and trust in friends capture more realistic aspects of trust than previous extreme measures. People’s familiarity with these situations allows for responses that better reflect everyday behavior.

Non-additive genetic variance estimates can be biased by sibling interactions. Competitive sibling interactions inflate estimates of D and occur when one twin’s high trait score lowers the score of the co-twin. This implies that if one twin is highly trusting, his or her co-twin becomes less trusting. It could be argued that if being too trusting has negative consequences, then one twin observing the other twin’s trustworthiness could lead them to be less trusting. However, as the co-twin is likely a very important relationship for the learning and building of trust, this explanation seems unlikely. In addition, there was no indication of sibling interaction in the variances of trust in MZ and DZ twin pairs.

Furthermore, GREML is agnostic to the pathways through which heritability is generated. Therefore, gene-environment correlations that inflate twin estimates might also be inflating DNA-based estimates. However, low DNA-based estimates suggest that if they do play a role, they are in fact small. Equally, GREML is not sensitive to the influence of cultural transmission, so the disparity between twin and DNA-based heritability estimates of trust continues to support cultural transmission as an explanation.

## Conclusion

In conclusion, despite high twin heritability estimates of 51–57%, the current findings suggest that common SNPs only account for a small amount of the variance. A large amount of the variance could be explained if the estimate tends toward the upper confidence interval; however, the current analysis lacks power to return a more accurate estimate. Gene–environment correlations and other genetic variants could partially explain this missing heritability gap. This finding is in line with DNA-based heritability estimates of the related trait of subjective well-being. Twin heritability estimates between this and previous studies vary, and therefore a greater understanding of the environments influencing trust and their interactions with genetic propensities would be the next step.

## Supplementary Material

To view supplementary material for this article, please visit http://dx.doi.org/10.1017/thg.2016.84

Supplementary Materials

## Figures and Tables

**Table 1:** Intraclass Correlations for Generalized Trust and Trust in Friends

	MZ twins	DZ twins
Generalized trust	0.34*** (*N* = 1,283 pairs)	0.11*** (*N* = 2,309 pairs)
Trust in friends	0.51*** (*N* = 811 pairs)	0.15*** (*N* = 1,258 pairs)

Note: *** *p* < .001. MZ = monozygotic twins; DZ = dizygotic twins.

**Table 2:** Model Fitting Results for Generalized Trust and Trust in Friends

		Testing against	-2LL	*df*	Par	χ^2^	Δ*df*	*p* value	AIC (*df*p)	BIC (ssa)
	Generalized trust									
G1	Saturated		6471.17	7,344	8	–	–	–	-8216.83	6521.28
G2	ACE	G1	6474.23	7,348	6	3.06	4	.55	-8221.78	6511.80
G3	**ADE**	**G1**	** 6472.91**	**7,348**	**6**	**1.74**	**4**	** .78**	** -8223.09**	** 6510.49**
G4	AE	G2	6474.23	7,349	5	0.00	1	1	-8223.78	6505.54
G5	AE	G3	6474.23	7,349	5	1.32	1	.25	-8223.78	6505.54
G6	CE	G2	6501.54	7,349	5	27.31	1	.00	-8196.46	6532.85
G7	DE	G3	6476.61	7,349	5	3.70	1	.05	-8221.40	6507.92
G8	E	G2	6618.25	7,349	4	144.02	1	.00	-8079.75	6643.30
G9	E	G3	6618.25	7,349	4	145.34	1	.00	-8079.75	6643.30
	Trust in friends									
F1	Saturated		29158.91	4,436	9	–	–	–	20286.91	29200.76
F2	ACE	F1	29170.00	4,439	6	11.09	3	.01	20292.00	29197.90
F3	**ADE**	**F1**	** 29160.75**	**4,439**	**6**	**1.84**	**3**	** .61**	**20282.75**	**29188.65**
F4	AE	F2	29170.00	4,440	5	0.00	1	1	20290.00	29193.25
F5	AE	F3	29170.00	4,440	5	9.25	1	.00	20290.00	29193.25
F6	CE	F2	29240.76	4,440	5	70.75	1	.00	20360.76	29 264
F7	DE	F3	29170.00	4,440	5	9.25	1	.00	20290.00	29185.4
F8	E	F2	29431.21	4,441	4	261.21	2	.00	20549.21	29449.81
F9	E	F3	29431.21	4,441	4	270.46	2	.00	20549.21	29449.81

Note: -2LL = minus 2 log likelihood; Par = number of estimated parameters; χ^2^ = chi-square (difference in -2LL); Δ*df* = difference in degrees of freedom; *p* = *p* value; AIC = Akaike’s information criteria (degrees of freedom penalty); BIC = Bayesian information criterion (sample size adjusted); G= generalized trust; F = trust in friends. Bold font indicates the best-fitting model.

**Table 3:** Standardized Parameter Estimates With 95% Confidence Intervals for the Best Fitting Model

	Parameter estimate		
	*a*^2^ [CI]	*d*^2^ [CI]	*e*^2^ [CI]
Generalized trust	0.35 [0.00, 0.60]	0.22 [0.00, 0.60]	0.43 [0.35, 0.52]
Trust in friends	0.14 [0.00, 0.36]	0.36 [0.13, 0.54]	0.50 [0.45, 0.55]

Note: *a*^2^ = estimate of additive genetic variance, *d*^2^ = estimate of non-additive genetic variance, *e*^2^ = estimate of non-shared environmental variance.

**Table 4:** SNPs Previously Associated With Trust Related Phenotypes

		Generalized trust		Trust in friends	
SNP	Previous association	*p* value	β effect size	*p* value	β effect size
rs4833624	Agreeableness (Kim et al., 2013)	.44	0.06	.49	0.46
rs254022	Agreeableness (Terracciano et al., 2010)	N/A	N/A	N/A	N/A
rs683276	Agreeableness (Terracciano et al., 2010)	N/A	N/A	N/A	N/A
rs12934132	Agreeableness (Kim et al., 2013)	.68	-0.04Δ	.72	0.28
rs9611312	Agreeableness (Kim et al., 2013)	.58	0.05	.31	0.84
rs2087017	Agreeableness (Kim et al., 2013)	.91	0.01	.09	-1.04Δ
rs16923100	Agreeableness (Kim et al., 2013)	.60	0.08Δ	.40	1.10Δ
rs11219218	Agreeableness (Kim et al., 2013)	.38	-0.07Δ	.46	0.57
rs1488467	Oxytocin (OXTR)	.81	0.04	.98	0.03
rs2268494	Oxytocin (OXTR)	.89	-0.02	.86	-0.21
rs237888	Oxytocin (OXTR)	.81	0.04	.70	0.50
rs237897	Oxytocin (OXTR)	.44	0.05	.74	-0.21
rs4686302	Oxytocin (OXTR)	.45	-0.09	.28	1.14
rs7632287	Oxytocin (OXTR)	.90	-0.01	.19	-0.93
rs9872310	Oxytocin (OXTR)	.47	0.07	.28	-0.97
rs3796863	CD38	.18	-0.10	.80	-0.17

Note: N/A = not applicable for those associated SNPs not available in the TEDS sample. No proxies were found for rs254022 or rs683276. The top half of the table includes SNPs previously associated in other studies. Here, Δ indicates an effect in the expected direction from Kim et al. (2013). In the lower half of the table, SNPs indicated as oxytocin (OXTR) are SNPs available in the oxytocin gene, which has previously been implicated, but no expected direction of effect is available for these SNPs.
