# Supplementary material for: Exploring the Genetic Etiology of Trust in Adolescents: Combined Twin and DNA Analyses
Source: Twin Res Hum Genet. Author manuscript; Available in PMC 2017 Jan 24. (PMC5260944; doi:10.1017/thg.2016.84)
Supplement: Supplementary Materials [file NIHMS71057-supplement-Supplementary_Materials.docx]

Twin Research and Human Genetics

**Exploring the genetic aetiology of trust in adolescents: Combined twin and DNA analyses**

Robyn E Wootton^1,2^, Oliver S P Davis^2^, Abigail L Mottershaw^1,2^, R Adele H Wang^1,2^, Claire M A Haworth^1,2^

1. School of Experimental Psychology, University of Bristol, Bristol, BS8 1TU, UK
2. MRC Integrative Epidemiology Unit, School of Social and Community Medicine, University of Bristol, BS8 2BN, UK

**Supplementary Materials**

1. Twin Analysis

*Supplementary Table S1: Proportion of twin groups by sex and zygosity.*

|  | Generalised Trust | | Trust in Friends | |
| --- | --- | --- | --- | --- |
|  | **N** | **%** | **N** | **%** |
| MZ Male | 1142 | 16 | 659 | 14 |
| MZ Female | 1477 | 20 | 1123 | 24 |
| DZ Male | 1060 | 14 | 606 | 13 |
| DZ Female | 1329 | 18 | 901 | 19 |
| DZ Opposite Sex | 2344 | 32 | 1390 | 30 |

*Note: MZ = Monozygotic or identical twins, DZ = Dizygotic or fraternal twins, N = number of individuals.*

2. Genome-Wide Association Study

DNA Collection and QC procedure are outlined in the main text. For the GWAS, the 700,000 high-quality genotyped SNPs were imputed to HapMap2 and HapMap3 reference panels using IMPUTE2 (for further information see (Davis et al., 2014)). GWAS analyses were run separately for each phenotype with age, sex and two principal components as covariates. Analyses were run using SNPTEST version 2.5.2 (<https://mathgen.stats.ox.ac.uk/genetics_software/snptest/snptest.html)>.

As discussed in the main text, no associations were found to be significant at the genome-wide level of significance. The 50 most significant SNPs for generalised trust and trust in friends are listed in Tables S2 and S3 respectively. The associations of previous candidate genes were also replicated and the results of these SNP associations are shown in Table 4 in main text.

*Generalised Trust*


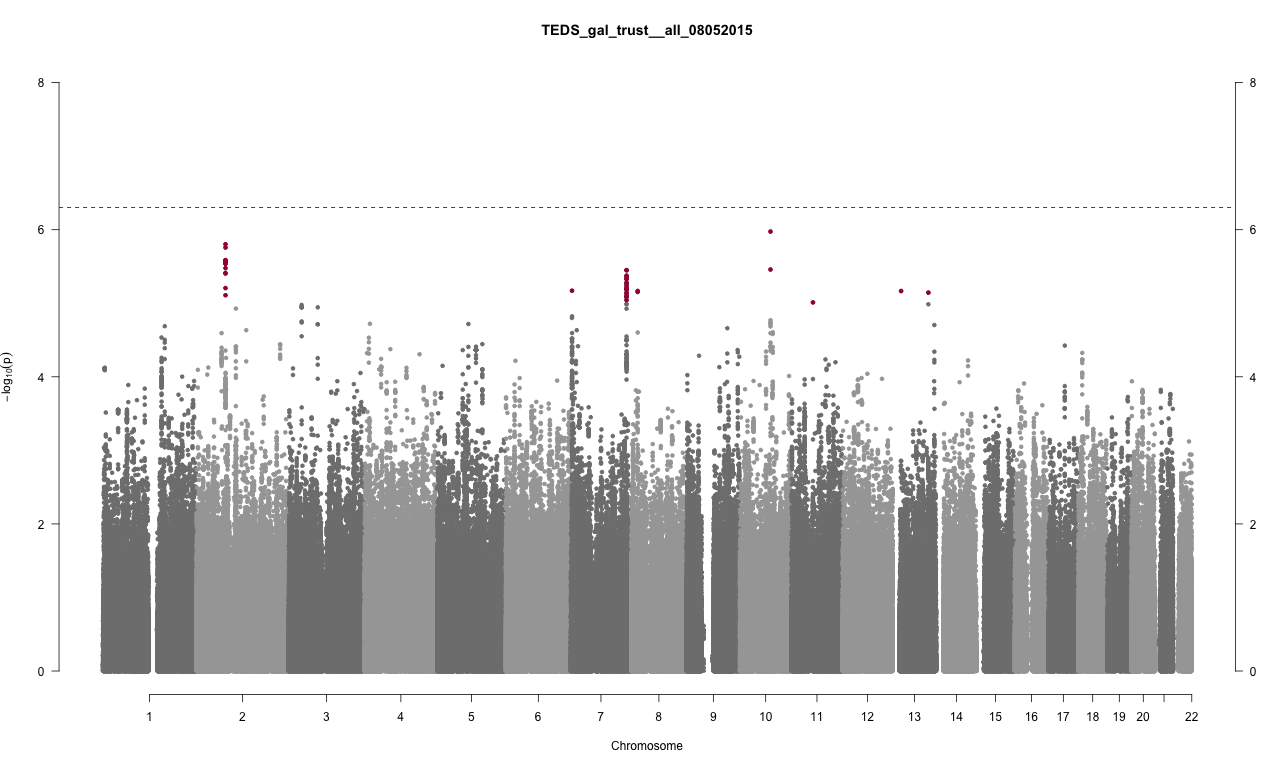


*Trust in Friends*


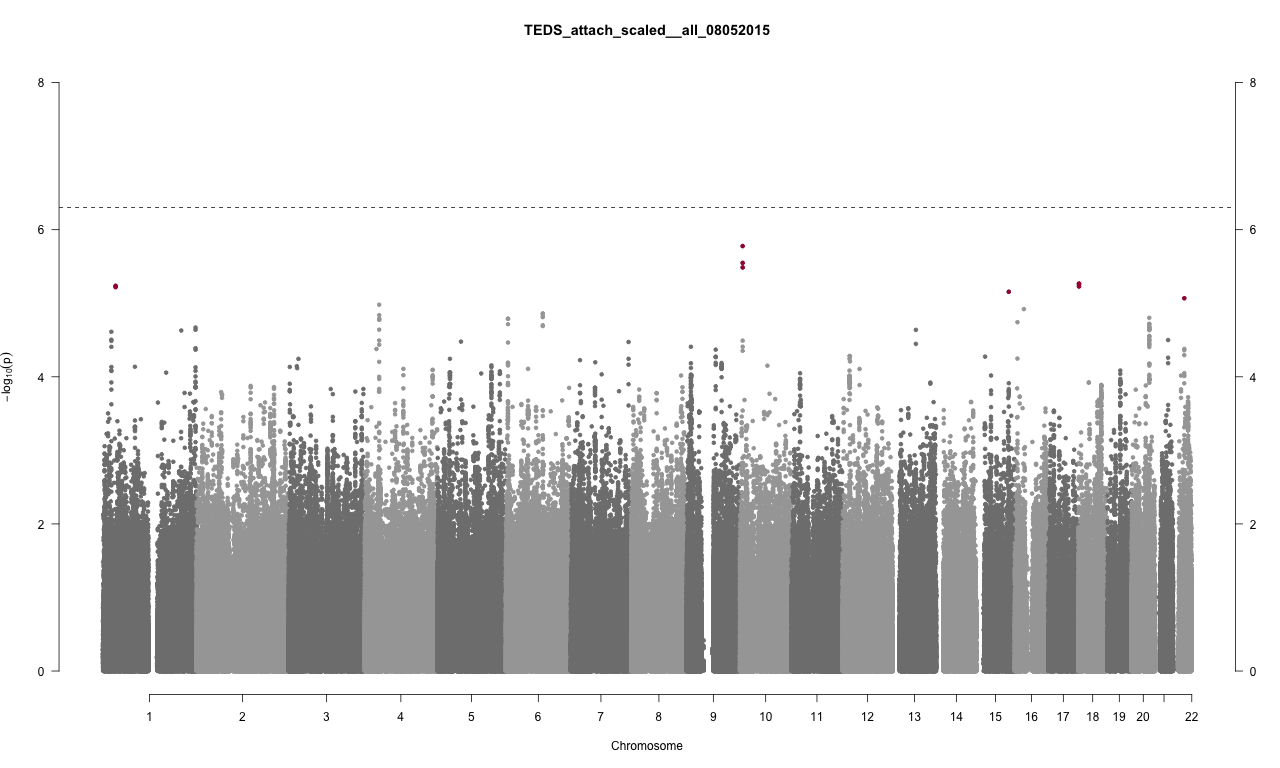


*Supplementary Figure S1: Genome-wide Manhattan Plots for Generalised Trust (N=1641) and Trust in Friends (N=1117). P-values were calculated on SNPTEST using a missing missing data likelihood score test. The horizontal line represents genome-wide significance (5 x 10^-8^). No SNPs at the genome-wide significance level were revealed. Red points represent SNPs with a p-value of less than 5 x 10^-6^. The 50 most significant SNPs for each are shown in Supplementary Tables 2 and 3.*

*Generalised Trust*


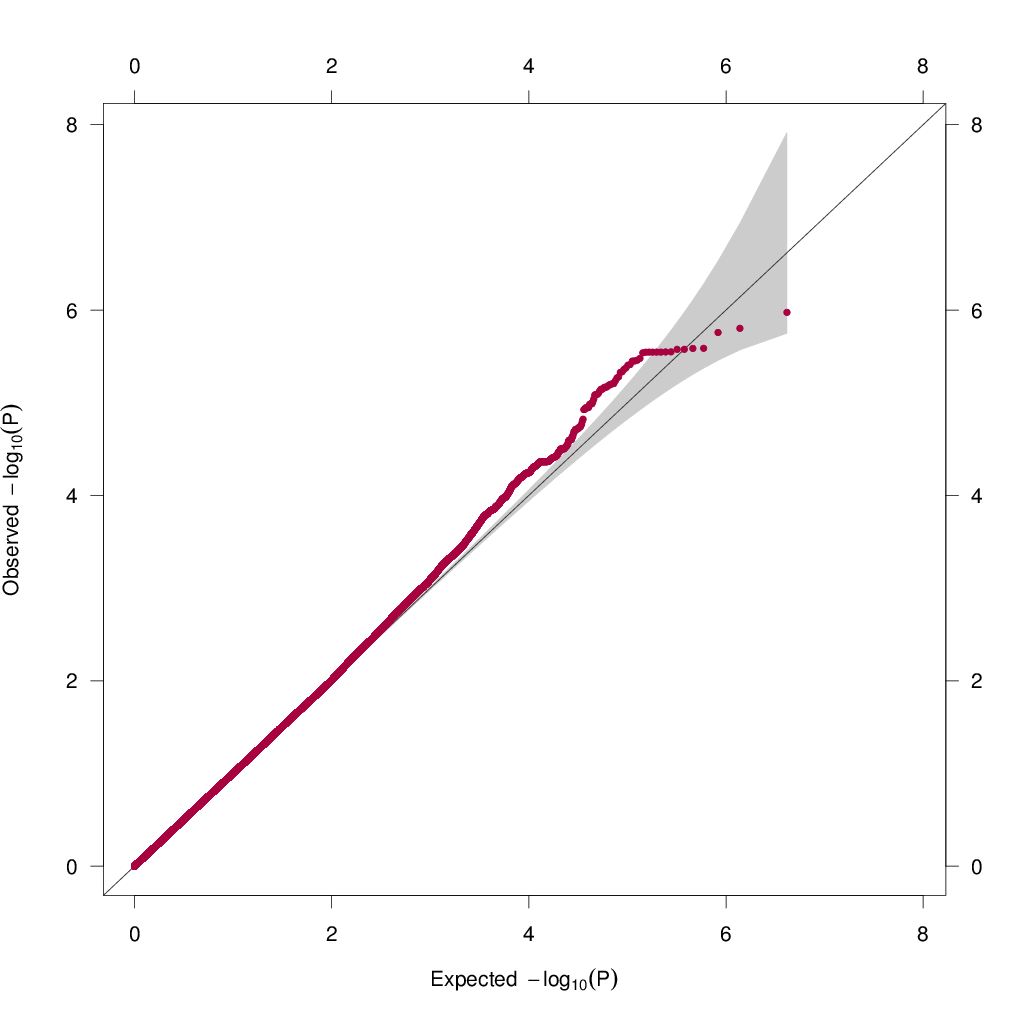


*Trust in Friends*


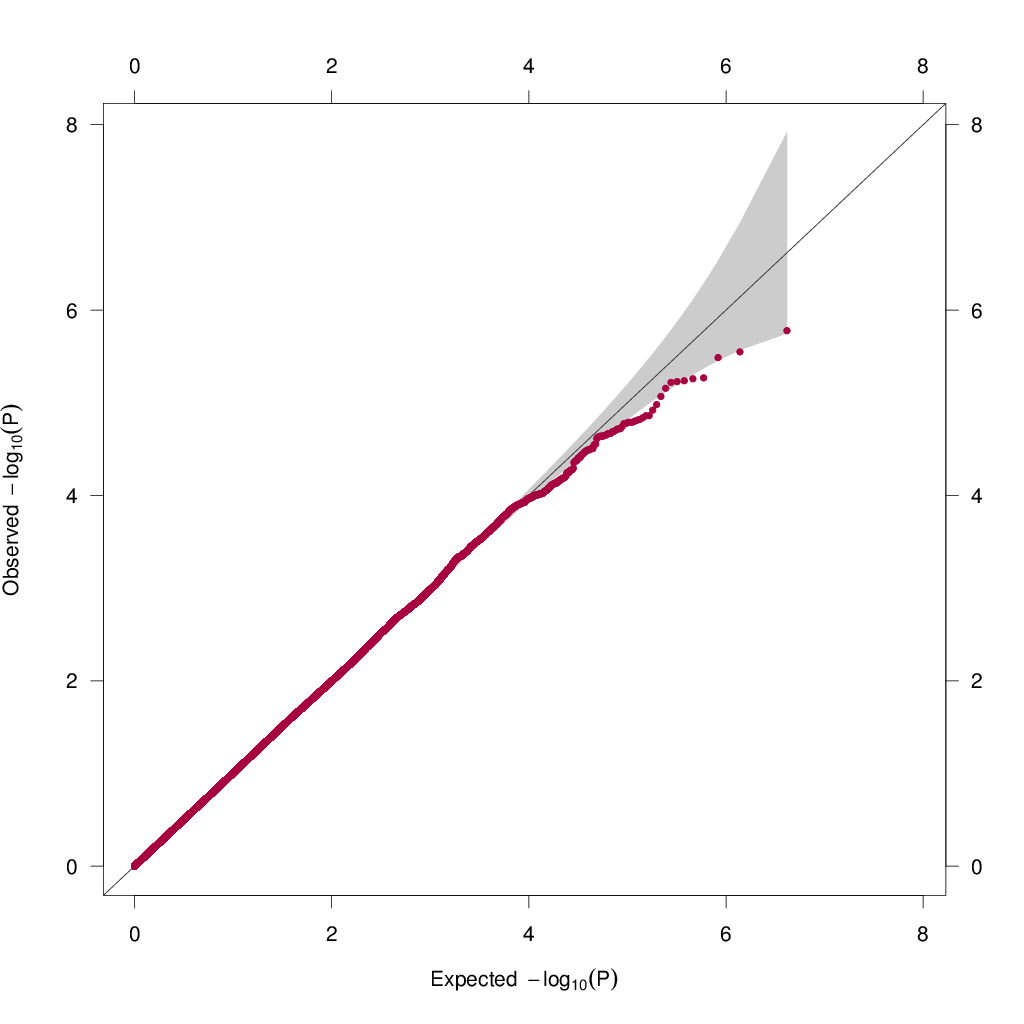


*Supplementary Figure S2: Quantile-quantile Plots for Generalised Trust (N=1641) and Trust in Friends (N=1117), based upon p-values calculated on SNPTEST using a missing missing data likelihood score test. The negative log_10_ of each SNP is plotted against what would be expected under the null (as indicated by the diagonal line). The grey area is a 95% confidence band on the null. The quantile-quantile plot of generalised trust does indicate some evidence of polygenic lift-off (many genes having small effects on the phenotype) where the line lifts above this grey area.*

*Supplementary Table S2: 50 most significant SNP associations for generalised trust.*

|  | rs Number | Chromosome | p-Value | β Effect Size |
| --- | --- | --- | --- | --- |
| 1 | rs10824657 | 10 | 1.06E-06 | -0.60 |
| 2 | rs2140571 | 2 | 1.58E-06 | 1.16 |
| 3 | rs1524216 | 2 | 1.75E-06 | 1.15 |
| 4 | rs425915 | 2 | 2.59E-06 | 1.14 |
| 5 | rs449418 | 2 | 2.60E-06 | -1.13 |
| 6 | rs6759874 | 2 | 2.66E-06 | -1.14 |
| 7 | rs956049 | 2 | 2.66E-06 | -1.14 |
| 8 | rs390810 | 2 | 2.83E-06 | -1.14 |
| 9 | rs317290 | 2 | 2.84E-06 | 1.13 |
| 10 | rs1608588 | 2 | 2.85E-06 | -1.14 |
| 11 | rs1372007 | 2 | 2.85E-06 | 1.14 |
| 12 | rs1524214 | 2 | 2.85E-06 | -1.14 |
| 13 | rs1404089 | 2 | 2.85E-06 | -1.14 |
| 14 | rs6753413 | 2 | 2.85E-06 | -1.14 |
| 15 | rs317288 | 2 | 2.90E-06 | 1.14 |
| 16 | rs1440883 | 2 | 3.33E-06 | -1.12 |
| 17 | rs2999469 | 10 | 3.48E-06 | 0.56 |
| 18 | rs10267232 | 7 | 3.55E-06 | -0.73 |
| 19 | rs6464815 | 7 | 3.55E-06 | -0.71 |
| 20 | rs10170802 | 2 | 3.88E-06 | 1.10 |
| 21 | rs2901742 | 2 | 3.94E-06 | 1.10 |
| 22 | rs1018991 | 7 | 4.22E-06 | -0.72 |
| 23 | rs826651 | 7 | 4.37E-06 | -0.73 |
| 24 | rs826650 | 7 | 4.64E-06 | -0.73 |
| 25 | rs826647 | 7 | 4.71E-06 | 0.73 |
| 26 | rs10228191 | 7 | 5.25E-06 | -0.73 |
| 27 | rs10264424 | 7 | 5.41E-06 | 0.73 |
| 28 | rs1528532 | 7 | 5.79E-06 | -0.79 |
| 29 | rs1660568 | 2 | 6.22E-06 | -1.08 |
| 30 | rs6973129 | 7 | 6.24E-06 | 0.71 |
| 31 | rs7780096 | 7 | 6.33E-06 | -0.71 |
| 32 | rs7779464 | 7 | 6.40E-06 | 0.71 |
| 33 | rs7778880 | 7 | 6.56E-06 | 0.71 |
| 34 | rs12700790 | 7 | 6.76E-06 | 0.80 |
| 35 | rs7005753 | 8 | 6.81E-06 | 0.50 |
| 36 | rs4145891 | 13 | 6.83E-06 | 0.67 |
| 37 | rs2239879 | 8 | 7.02E-06 | 0.52 |
| 38 | rs7321687 | 13 | 7.17E-06 | -0.51 |
| 39 | rs10265626 | 7 | 7.17E-06 | 0.72 |
| 40 | rs1585815 | 7 | 7.40E-06 | -0.71 |
| 41 | rs12473082 | 2 | 7.79E-06 | -1.06 |
| 42 | rs826656 | 7 | 8.07E-06 | -0.71 |
| 43 | rs826658 | 7 | 8.15E-06 | 0.71 |
| 44 | rs826659 | 7 | 8.17E-06 | -0.71 |
| 45 | rs826661 | 7 | 8.27E-06 | 0.71 |
| 46 | rs700313 | 7 | 9.09E-06 | 0.71 |
| 47 | rs10501366 | 11 | 9.74E-06 | 0.54 |
| 48 | rs826655 | 7 | 1.03E-05 | -0.70 |
| 49 | rs826657 | 7 | 1.03E-05 | -0.71 |
| 50 | rs9516371 | 13 | 1.03E-05 | -0.48 |

*Note: None of these SNPs are previous candidate associations.*

*Supplementary Table S3: 50 most significant SNP associations for trust in friends.*

|  | rs Number | Chromosome | p-Value | β Effect Size |
| --- | --- | --- | --- | --- |
| 1 | rs4747368 | 10 | 1.67E-06 | 0.21 |
| 2 | rs881703 | 10 | 2.83E-06 | -0.21 |
| 3 | rs2050718 | 10 | 3.26E-06 | -0.21 |
| 4 | rs11080909 | 18 | 5.40E-06 | -0.20 |
| 5 | rs1510022 | 18 | 5.52E-06 | -0.20 |
| 6 | rs6677917 | 1 | 5.80E-06 | -0.36 |
| 7 | rs6505983 | 18 | 5.92E-06 | 0.20 |
| 8 | rs10753296 | 1 | 6.03E-06 | -0.37 |
| 9 | rs7173322 | 15 | 6.98E-06 | 0.19 |
| 10 | rs5997872 | 22 | 8.56E-06 | -0.25 |
| 11 | rs6824769 | 4 | 1.05E-05 | 0.19 |
| 12 | rs1557811 | 16 | 1.20E-05 | 0.32 |
| 13 | rs1858390 | 6 | 1.38E-05 | 0.67 |
| 14 | rs1398160 | 6 | 1.38E-05 | -0.67 |
| 15 | rs7664107 | 4 | 1.45E-05 | -0.18 |
| 16 | rs9372186 | 6 | 1.51E-05 | -0.67 |
| 17 | rs7752082 | 6 | 1.54E-05 | 0.66 |
| 18 | rs6019574 | 20 | 1.58E-05 | 0.21 |
| 19 | rs6923842 | 6 | 1.62E-05 | -0.23 |
| 20 | rs6924018 | 6 | 1.63E-05 | 0.23 |
| 21 | rs1115259 | 4 | 1.63E-05 | -0.18 |
| 22 | rs12641669 | 4 | 1.67E-05 | -0.18 |
| 23 | rs12642243 | 4 | 1.68E-05 | 0.18 |
| 24 | rs12051021 | 16 | 1.81E-05 | -0.63 |
| 25 | rs4810906 | 20 | 1.91E-05 | 0.21 |
| 26 | rs4810907 | 20 | 1.92E-05 | -0.21 |
| 27 | rs7747282 | 6 | 1.93E-05 | -0.24 |
| 28 | rs4839854 | 6 | 1.99E-05 | 0.63 |
| 29 | rs6939248 | 6 | 2.04E-05 | 0.63 |
| 30 | rs2426127 | 20 | 2.05E-05 | -0.20 |
| 31 | rs6019543 | 20 | 2.13E-05 | -0.20 |
| 32 | rs7518917 | 1 | 2.14E-05 | 0.20 |
| 33 | rs4810886 | 20 | 2.15E-05 | 0.21 |
| 34 | rs6125522 | 20 | 2.19E-05 | -0.20 |
| 35 | rs3092153 | 20 | 2.25E-05 | -0.20 |
| 36 | rs1473718 | 20 | 2.26E-05 | -0.20 |
| 37 | rs974734 | 4 | 2.29E-05 | 0.18 |
| 38 | rs1413884 | 1 | 2.29E-05 | 0.20 |
| 39 | rs1473717 | 20 | 2.30E-05 | -0.20 |
| 40 | rs1483703 | 13 | 2.30E-05 | -0.17 |
| 41 | rs2426105 | 20 | 2.33E-05 | 0.20 |
| 42 | rs12036211 | 1 | 2.35E-05 | 0.18 |
| 43 | rs6701399 | 1 | 2.44E-05 | 0.18 |
| 44 | rs4810905 | 20 | 2.80E-05 | 0.20 |
| 45 | rs3795086 | 20 | 2.80E-05 | -0.20 |
| 46 | rs4810910 | 20 | 2.89E-05 | 0.20 |
| 47 | rs4810904 | 20 | 3.13E-05 | 0.20 |
| 48 | rs10917312 | 1 | 3.13E-05 | 0.18 |
| 49 | rs6012580 | 20 | 3.13E-05 | -0.21 |
| 50 | rs1109848 | 21 | 3.17E-05 | -0.20 |

*Note: None of these SNPs are previous candidate associations.*

**References**

Davis, O. S. P., Band, G., Pirinen, M., Haworth, C. M. A., Meaburn, E. L., Kovas, Y., … Spencer, C. C. A. (2014). The correlation between reading and mathematics ability at age twelve has a substantial genetic component. *Nature Communications*, *5*, 4204. http://doi.org/10.1038/ncomms5204
